# Binding interaction of a *gamma*-aminobutyric acid derivative with serum albumin: an insight by fluorescence and molecular modeling analysis

**DOI:** 10.1186/s40064-016-2752-x

**Published:** 2016-07-19

**Authors:** Uttam Pal, Sumit Kumar Pramanik, Baisali Bhattacharya, Biswadip Banerji, Nakul C. Maiti

**Affiliations:** Structural Biology and Bioinformatics Division, Council of Scientific and Industrial Research (CSIR)-Indian Institute of Chemical Biology (IICB), Kolkata, West Bengal India; Chemistry Division, Council of Scientific and Industrial Research (CSIR)-Indian Institute of Chemical Biology (IICB), Kolkata, West Bengal India

**Keywords:** Serum albumin, Fluorescence, GABA, Molecular docking, Molecular dynamics

## Abstract

*gamma*-Aminobutyric acid (GABA) is a naturally occurring inhibitory neurotransmitter and some of its derivatives showed potential to act as neuroprotective agents. With the aim of developing potential leads for anti-Alzheimer’s drugs, in this study we synthesized a novel GABA derivative, methyl 4-(4-((2-(*tert*-butoxy)-2-oxoethyl)(4-methoxyphenyl)amino)benzamido)butanoate by a unique method of Buchwald–Hartwig cross coupling synthesis; with some modification the yield was significant (97 %) and spectroscopic analysis confirmed that the compound was highly pure (98.8 % by HPLC). The druglikeness properties such as logP, logS, and polar surface area were 3.87, −4.86 and 94.17 Å^2^ respectively and it satisfied the Lipinski’s rule of five. We examined the binding behavior of the molecule to human serum albumin (HSA) and bovine serum albumin (BSA) which are known as universal drug carrier proteins. The molecule binds to the proteins with low micromolar efficiency and the calculated binding constants were 3.85 and 2.75 micromolar for BSA and HSA, respectively. Temperature dependent study using van’t Hoff equation established that the binding was thermodynamically favorable and the changes in the Gibb’s free energy, ΔG for the binding process was negative. However, the binding of the molecule to HSA was enthalpy driven and the change of enthalpy (ΔH) was −10.63 kJ/mol, whereas, the binding to BSA was entropy driven and the change in entropy ΔS was 222 J/mol. The molecular docking analysis showed that the binding sites of the molecule lie in the groove between domain I and domain III of BSA, whereas it is within the domain I in case of HSA, which also supported the different thermodynamic nature of binding with HSA and BSA. Molecular dynamics analysis suggested that the binding was stable with time and provided further details of the binding interaction. Molecular dynamics study also highlighted the effect of this ligand binding on the serum albumin structure.

**Electronic supplementary material:**

The online version of this article (doi:10.1186/s40064-016-2752-x) contains supplementary material, which is available to authorized users.

## Background

*gamma*-Aminobutyric acid (GABA) plays an important role as an inhibitory neurotransmitter of the central nervous system (Gajcy et al. 2010). Impaired secretion of GABA is associated with several important neurological disorders such as Parkinson’s (Kleppner and Tobin 2001) and Alzheimer’s disease (Jo et al. 2014) and other psychiatric disorders (Nutt and Malizia 2001). Amyloid-β (Aβ) is an intrinsically disordered protein and therefore, it does not have an ordered native structure under physiological condition (Lu et al. 2013). However, the structure of Aβ evolves or gets stabilized as it forms higher order aggregates (Lu et al. 2013) such as oligomers and thread like elongated fibril with cross beta sheet structure. Therefore, the binding sites on Aβ also evolve with the process of aggregation. Drugs that bind to amyloid beta at different stages of aggregation have been developed to arrest the further growth of oligomers (Padayachee and Whiteley 2011; Huy et al. 2013; Richard et al. 2013).

New GABA derivatives can be considered as potential drugs in the treatment of neurodegenerative disorders (Gajcy et al. 2010). The efficacy of GABA can be highly potentiated by benzodiazopines (Nutt and Malizia 2001). Recently there has been increasing interest in synthesizing new GABA derivatives, thus, these compounds could be the potential lead molecules in the development of anti-Alzheimer’s drugs to target Aβ peptide or its assembly structures (Jiang et al. 2013).

To realize the interaction pattern of the newly synthesized GABA derivative, methyl 4-(4-((2-(*tert*-butoxy)-2-oxoethyl)(4-methoxyphenyl)amino)benzamido)butanoate, in hydrophobic protein cavities we explored both the quantitative and qualitative aspects of the interaction and incorporation of the compound into the binding pockets of serum albumin using fluorescence, molecular docking and molecular dynamics analysis. This compound is very similar to a series of molecules which were previously tested by Dr. Banerji’s group for their anti-Alzheimer’s activity (Sanphui et al. 2013).

The incorporation and investigation of the molecule inside the hydrophobic protein environment was monitored following the changes in the intrinsic tryptophan fluorescence of the protein molecule. Intrinsic protein fluorescence originating from tryptophan and tyrosine residues provides ample information about the local environment, the changes in protein conformation and the interaction of a protein with a drug molecule (Möller and Denicola 2002). Perturbation in fluorescence intensity also provides significant insight into the interaction pattern of the molecules. Binding parameters were measured from fluorescence quenching in the presence of the compound and related thermodynamic parameters were obtained by measuring the effect of temperature on binding constant.

In addition, to find out the interaction of the drug molecule at atomic level, molecular docking and dynamics analysis were carried out. Molecular docking is a robust and efficient computational technique to understand the structure activity relationship of a drug-like molecule with a target protein (Jorgensen 2004; Morris and Lim-Wilby 2008; Meng et al. 2011). The binding sites of the GABA derivative on serum albumins, interacting residues and the type of interactions were probed by molecular docking analysis. Molecular dynamics study further ascertained the stability of binding and highlighted the specific interactions over a time period.

## Methods

### Chemicals

Bovine and human serum albumins were purchased from Sigma-Aldrich Corporation (St. Louis, MO, USA). Tris–HCl and Urea were also purchased from Sigma-Aldrich. All the samples were prepared in 20 mM Tris–HCl buffer of pH 7.0. Deionized and triple distilled water was used for preparing buffer solution that was passed through 0.22 µm pore size Millipore filters (Millipore India Pvt. Ltd., Bangalore, India).

All air and water sensitive reactions were carried out in oven dried glassware under nitrogen atmosphere using standard manifold techniques. All the chemicals were purchased from Acros organics and Sigma-Aldrich, and used without further purification unless otherwise stated. Compounds that are not described in the experimental part were synthesized according to the literature procedures. Solvents were freshly distilled by standard procedures prior to use. Flash chromatography was performed on silica gel (Merck, 100–200 mesh) with the indicated eluant. All ^1^H and ^13^C-NMR spectra were recorded on a Bruker 600 MHz spectrometer. For ^1^H NMR, tetramethylsilane (TMS) served as internal standard (δ = 0) and data are reported as follows: chemical shift, integration, multiplicity (s = singlet, d = doublet, t = triplet, q = quartet, m = multiplet) and coupling constant(s) in Hz. For ^13^C NMR, TMS (δ = 0) or CDCl_3_ (δ = 77.26) was used as internal standard and spectra were obtained with complete proton decoupling.

### Procedure to synthesize the GABA derivative

**Scheme 1:**
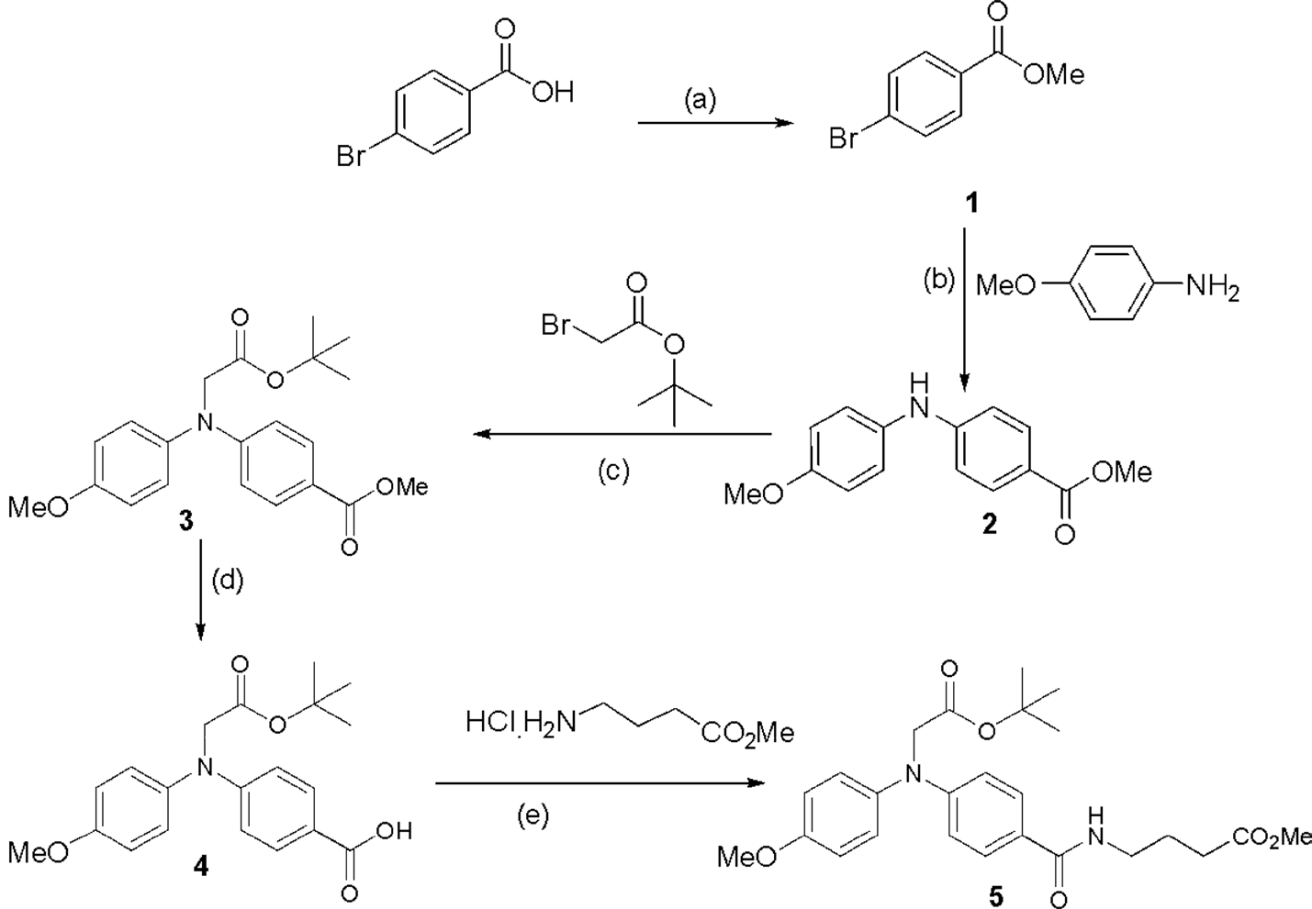
Reagent and conditions: **a** Conc. H_2_SO_4_, MeOH, 0 °C to r.t., 6 h. **b** Palladium(II) acetate (0.05 equiv.), xantphos (0.1 equiv.) and cesium carbonate (3 equiv.), 80 °C, 4 h. **c** Potassium *tert*-butoxide (1.2 equiv.), DMF, 0 °C to r.t., 12 h. **d** Lithium hydroxide (3 equiv.), MeOH–water (5:1), r.t., 2 h. **e** EDC.HCl (3.0 equiv.), HOBT (2.5 equiv.), TEA (6 equiv.), 0 °C to r.t., 1.5 h

Synthesis of the compound methyl 4-(4-((2-(*tert*-butoxy)-2-oxoethyl)(4-methoxyphenyl)amino)benzamido)butanoate was carried out following Scheme 1. Detailed procedure and characteristic data are given in the supporting information.

### Absorption spectroscopy

Ground-state absorption spectra were recorded with a Shimadzu UV-2401PC Spectrometer. 1 cm path-length quartz cuvette was used and 250–450 nm wavelength range was scanned.

### Fluorescence emission spectroscopy

The steady-state fluorescence emission and excitation spectra were recorded with a Cary Eclipse Fluorescence Spectrophotometer. The emission spectra of serum albumins were obtained by exciting the samples at the wavelength 295 nm. In all the cases, the excitation and emission slit widths were kept at 5 nm each. Protein fluorescence spectra as a function of ligand concentration was recorded by a simple titration method (Banerjee et al. 2012, 2013; Ray et al. 2012).

### Determination of binding constants

The intrinsic fluorescence of protein was measured as a function of ligand concentration. The protein concentration was kept at 0.5 µM and the ligand concentration was varied from 0 to 5.5 µM. Small dilution error due to the titration was ignored. For the titration experiment, we used 100 µM stock solution of compound 5 in same buffer from which we added 10 µL compound 11 times to 2000 µL protein solution amounting to a maximum of 5.5 % dilution. Fluorescence intensities at maximum emission wavelength were recorded as a function of ligand concentration. To derive the binding parameters, data were analyzed using the non-linear Langmuir isotherm (Banerji et al. 2013):

1 $$\Delta {\text{F}} =\Delta {\text{F}}_{ \hbox{max} } \,*\,[{\text{Q}}]/({\text{K}}_{\text{d}} + [{\text{Q}}])$$

where ΔF is the difference in fluorescence in the absence and presence of the quencher at concentration [Q], ΔF_max_ is the maximum possible change in the fluorescence intensity, *K*_*d*_ is the binding dissociation constant. The non-linear equation was fitted to the data using Wolfram Mathematica 9. Stern–Volmer quenching constant or the binding affinity constant, K_a_ was determined as a reciprocal of *K*_*d*_ (Banerji et al. 2013).

### Binding thermodynamics

*K*_*d*_ values were determined as a function of temperature and the thermodynamic parameters of binding were obtained by fitting van’t Hoff equation (Banerjee et al. 2012; Ray et al. 2012) to the data:

2 $$\ln {\text{K}}_{\text{eq}} = -\Delta {\text{H}}^\circ /{\text{RT}} +\Delta {\text{S}}^\circ /{\text{R}}$$

where K_eq_ is the equilibrium constant (here the Stern–Volmer quenching constant) of binding at corresponding temperature T, and R is the gas constant. The equation gives the standard enthalpy change (ΔH°) and standard entropy change (ΔS°) on binding. The free energy change (ΔG°) has been estimated from the following relationship (Banerjee et al. 2012; Ray et al. 2012):

3 $$\Delta {\text{G}}^\circ =\Delta {\text{H}}^\circ - {\text{T}}\Delta {\text{S}}^\circ$$

### Lipophilicity and solubility calculations

Lipophilicity in terms of calculated logP (clogP) and solubility in terms of calculated logS (clogS) were determined at Virtual Computational Chemistry Laboratory server (http://www.vcclab.org/lab/alogps/) (Tetko et al. 2005). Polar surface area was calculated with a 1.4 Å radius probe size.

### Molecular docking

Molecular docking experiments were performed using four different algorithms: AutoDock Vina (Trott and Olson 2010), AutoDock 4.2 (Morris et al. 2009), PatchDock/FireDock (Schneidman-Duhovny et al. 2005; Mashiach et al. 2008) and SwissDock (Grosdidier et al. 2011). BSA (PDB: 3V03) (Majorek et al. 2012) and HSA (PDB: 4L8U) (Bhattacharya et al. 2000) structural information was obtained from Protein Data Bank (Berman et al. 2000). Protein structures were chosen based on the validation report provided by wwPDB at the PDB website (Read et al. 2011; Gore et al. 2012). All the hetero atoms and water and multiple subunits were removed from the PDB structures and the missing side chain residues for BSA were modeled at PDB_hydro web server (Azuara et al. 2006). The ligand structures were drawn in Avogadro (Hanwell et al. 2012) and geometry optimized *in vaccuo* using the steepest descent followed by conjugate gradient algorithms in UFF forcefield as implemented in Avogadro.

AutoDockTools (Morris et al. 2009) was used to prepare the ligand and proteins For the docking in AutoDock 4.2 and AutoDock Vina. Polar hydrogen atoms and Gasteiger charges were added to the proteins and the ligand. All the rotatable bonds in the ligand were set free. No flexibility was added to the protein side chains. The whole protein was placed in the center of a simulation box. The box dimension was 87 × 66 × 80 cubic angstroms for BSA and 87 × 66 × 73 cubic angstroms for HSA. Grid point spacing of 0.775 Å was used for docking in AutoDock 4.2, while the grid point spacing for AutoDock Vina was 1 Å. Genetic algorithm was run (ga_run) 100 times to generate a statistically significant number of docked poses (Alam et al. 2012). All the other parameters were kept constant. AutoDock Vina results were rendered in PyMOL and AutoDock 4.2 results were rendered in MGLTools.

Docking was also carried out at two different web servers: SwissDock and PatchDock/FireDock. SwissDock results were rendered in UCSF Chimera (Pettersen et al. 2004). PatchDock does not consider ligand flexibility, therefore, best poses of the ligand obtained by AutoDock 4.2, Vina and SwissDock were used as input ligand orientation for docking with PatchDock. 10 Best PatchDock results were further refined by FireDock web interface. FireDock results were rendered in PyMOL.

### Molecular dynamics

Molecular dynamics (MD) analysis was carried out in Schrodinger Maestro Molecular Modeling environment (academic release 2015-4). 12 ns dynamics were carried out for the protein ligand complexes and for the proteins as well, in SPC water environment using Desmond (Bowers et al. 2006) molecular dynamics program implemented in Schrodinger Maestro. The proteins or the complexes were placed in the center of the simulation box with periodic boundary conditions. The periodic boundary box dimensions are given in the supporting information (Additional file 1: Table S1). The whole systems were charge neutralized using sodium ions. MD was run in OPLS 2005 force field (Banks et al. 2005). Five step relaxation protocol was used starting with Brownian dynamics for 100 ps with restraints on solute heavy atoms at NVT (with T = 10 K) followed by 12 ps of dynamics with restraints at NVT (T = 10 K) and then at NPT (T = 10 K) using Berendsen method. Then the temperature was raised to 300 K for 12 ps followed by 24 ps relaxation step without restraints on the solute heavy atoms. The production MD was run at NPT with T = 300 K for 12,000 ps. The molecular dynamics output was rendered in Schrodinger Maestro Suite.

## Results and discussion

### Absorbance and fluorescence of the GABA derivative

Molecular structure of compound 5 is shown in Scheme 1. Additional file 1: Figure S1 shows the absorption spectrum of the GABA derivative. Due to the presence of conjugate systems, it showed absorption in UV region (below 300 nm). However, the absorbance was very weak. The compound is non-fluorescent in nature.

### Interaction with albumins

**Fig. 1:**
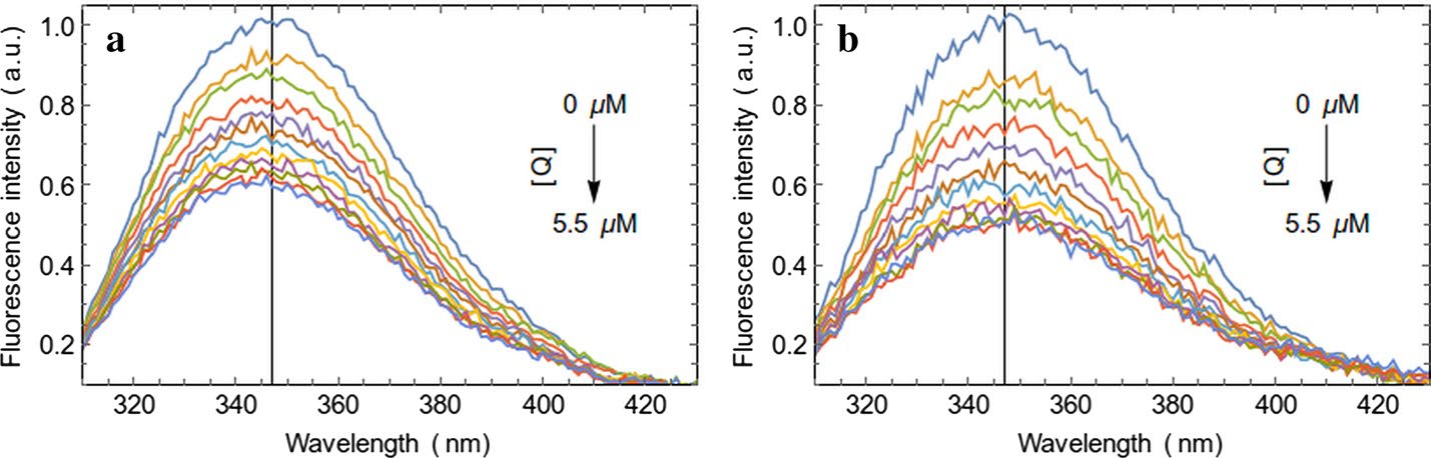
Fluorescence quenching of BSA and HSA with the GABA derivative. Serum albumin concentration was kept constant at 0.5 μM (in 20 mM Tris–HCl buffer of pH 7.0) and the ligand concentration was varied from 0 to 5.5 μM. **a** Spectral changes showing the quenching of intrinsic fluorescence emission of BSA as a function of ligand concentration. **b** Quenching of intrinsic fluorescence of HSA in presence of ligand. These two spectra were recorded at room temperature

**Fig. 2:**
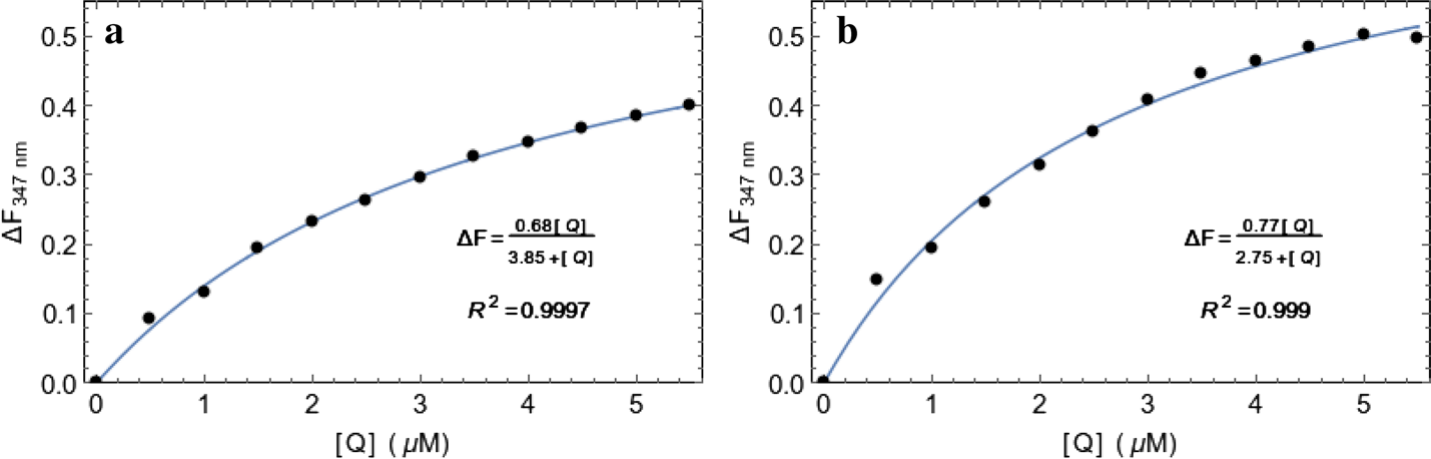
Determination of binding constants of the compound with serum albumins. **a** The changes in fluorescence intensity at the maximum emission wavelength and the fitted Langmuir isotherm for the determination of binding constant with BSA. **b** Langmuir isotherm for HSA binding

The fluorescence intensity of BSA and HSA decreased gradually with the increasing concentration of ligand (Fig. 1). Thus, the quenching of the intrinsic tryptophan fluorescence of serum albumins by the GABA derivative indicates its binding to the proteins. Figure 2 shows the Langmuir isotherm (Eq. 1) fitted to the quenching data for the determination of binding constants. The binding dissociation constants were found to be in the low micromolar concentration range (Table 1). Ligand shows a negligible absorbance at 295 nm wavelength. However, the experiments were carried out at very low concentrations of protein and ligand to avoid the inner filter effect.

**Table 1:** The K_d_ and K_a_ values for the binding of the GABA derivative to serum albumins as determined by the fluorescence quenching experiments at room temperature

Protein	K_d_ (M)	K_a_ (M^−1^)
BSA	3.85 × 10^−6^	2.60 × 10^5^
HSA	2.75 × 10^−6^	3.64 × 10^5^

### Thermodynamics of serum albumin binding

**Fig. 3:**
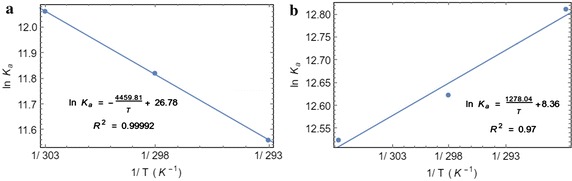
Determination of thermodynamic parameters of binding from van’t Hoff’s plot. **a** Decrease in the binding equilibrium constant with the decreasing temperature (303, 298 and 293 K) for the interaction with BSA and the fitted van’t Hoff equation. **b** Increase in the binding equilibrium constant with the decreasing temperature (308, 298 and 288 K) for the interaction with HSA and the fitted van’t Hoff equation

Equilibrium constant of a reaction changes with the temperature (Fig. 3), which is explained by van’t Hoff’s equation. The standard enthalpy and standard entropy changes for the reaction can also be obtained from van’t Hoff’s equation. The temperature depended fluorescence quenching study showed that the association of compound 5 with serum albumins is thermodynamically favorable, which is evident from the decrease in Gibbs free energy (Table 2). However, the binding with HSA was found to be enthalpy driven (negative ΔH°) whereas the binding with BSA was entropy driven (positive ΔS°). It suggests that, despite the structural similarity between the two proteins, the interactions with HSA are thermodynamically different from those with BSA. The similar trend was also observed for the binding of a naphthalene based fluorescent compound we previously reported (Pal et al. 2015). The molecule under investigation has structural similarity with the previously reported molecule, however, it contains a phenyl substituent instead of a naphthyl group.

**Table 2:** Thermodynamics of the GABA derivative binding to serum albumins

Serum albumins	ΔG° (kJ mol^−1^) at 25 °C	ΔH° (kJ mol^−1^)	ΔS° (J mol^−1^ K^−1^)
BSA	−29.27	37.08	222.67
HSA	−31.34	−10.63	69.5

### Drug like properties of the GABA derivative

The molecular properties of the compound such as clogP, clogS, and polar surface area (Bickerton et al. 2012) are listed in Table 3. The clogP value of a compound is the logarithm of its partition coefficient between n-octanol and water. It is a well established measure of the compound’s lipophilicity, which influences its behaviour in a range of biological processes such as solubility, membrane permeability, lack of selectivity and non-specific toxicity (Alam et al. 2011). It has been shown for compounds to have a reasonable probability of being well absorbed, their logP value must not be greater than 5.0 (Lipinski et al. 1997). Besides, the aqueous solubility of a compound is also defined by logS, which significantly affects its absorption and distribution characteristics. Typically, a low solubility goes along with a bad absorption. Most of the drugs on the market have an estimated logS value of about −4. Table 3 lists the polar surface area of the compound as well, which should be less than 140 Å^2^ for a drug molecule (Lipinski et al. 1997). Apart from lipophilicity/solubility and the polar surface area, the molecular weight and the number of hydrogen bond acceptor/donor in the compound also follow the Lipinski’s rule of five (Lipinski et al. 1997).

**Table 3:** Molecular properties of the compound

Properties	Values
logP^a^	3.87 ± 0.53
logS	−4.86
Polar surface area	94.17 Å^2^
Lipinski’s rule of five	Yes

^a^The data represent mean ± SD

### Molecular modeling provides insight into the interaction with serum albumins

**Fig. 4:**
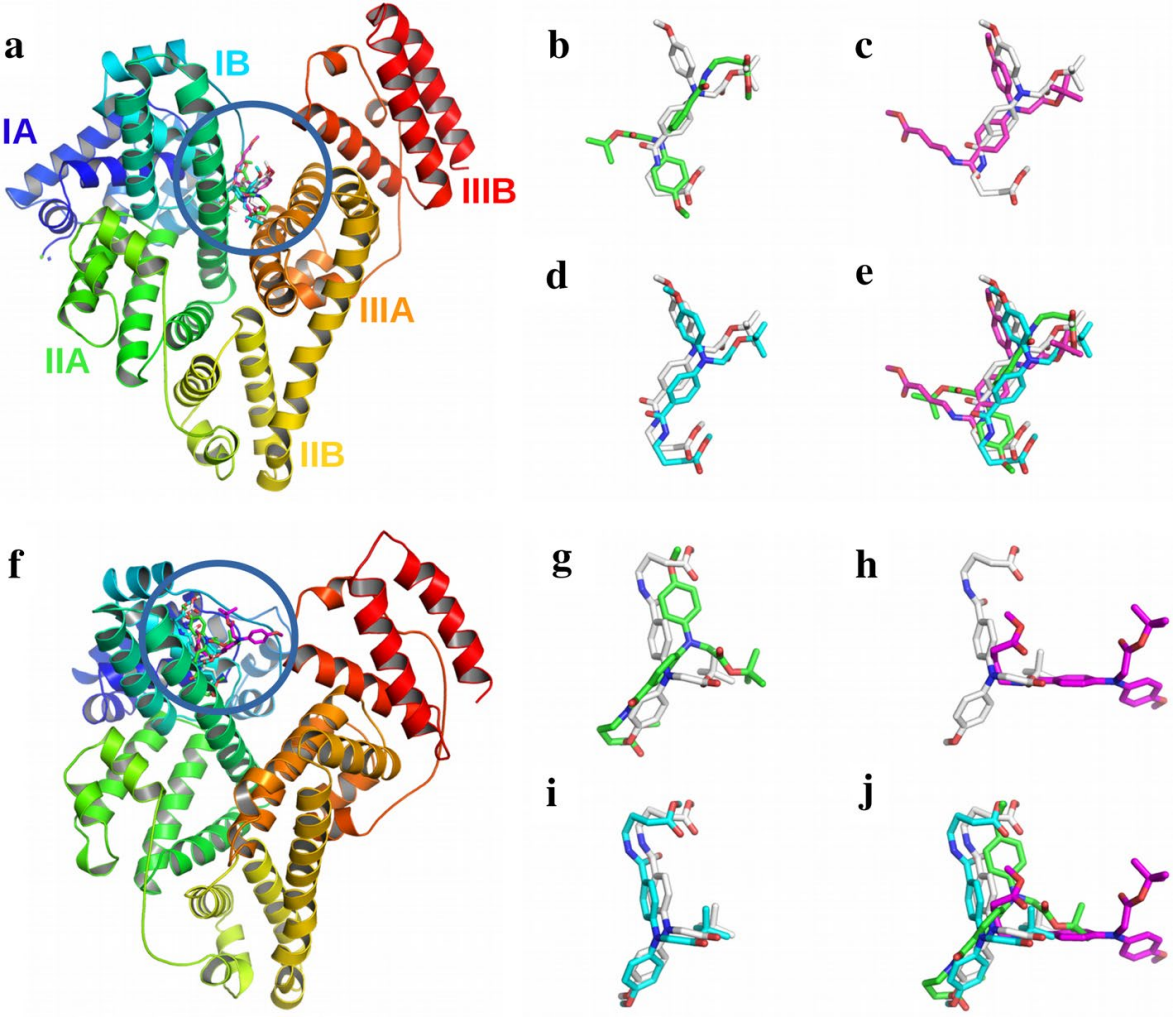
Interaction of the GABA derivative with serum albumins as obtained by molecular modeling. **a** Binding site of compound 5 on BSA. **b** Comparison of the best docked conformations with BSA as obtained by AutoDock Vina and AutoDock 4.2. **c** Comparison of Vina and SwissDock output for compound 5 binding with BSA. **d** PatchDock/FireDock shows Vina output has the best *shape* complementarity with BSA. **e** Overlap of best docked conformations of compound 5 with BSA. **f** Binding site of compound 5 on HSA. **g** Comparison of the best HSA binding poses as obtained by AutoDock Vina and AutoDock 4.2. **h** Comparison of Vina and SwissDock output for compound 5 binding with HSA. **i** PatchDock/FireDock showing Vina output has the best shape complementarity with HSA. **j** Overlap of best docked conformations of compound 5 with HSA. Proteins are shown in cartoon diagram and the ligands in stick model. The protein is *colored* in rainbow from N to C terminal. The three domains of serum albumin are marked with I–III. Standard *color* representation is used to denote the elements, H, N and O, in ligand. Ligand C in Vina, AutoDock 4.2, PatchDock/FireDock and SwissDock ouput are *colored* in *white*, *green*, *cyan* and *magenta*, respectively

It has been established that serum albumin proteins have at least seven hydrophobic grooves on their surface that provide a unique microenvironment and act as universal receptors for many drug molecules (Curry et al. 1998; Simard et al. 2006; Reichenwallner and Hinderberger 2013). Binding to these hydrophobic sites increases the solubility of hydrophobic ligands in plasma and modulates their delivery to cells. The precise architecture of the binding pockets is known from several crystallographic and NMR spectroscopic studies (Curry et al. 1998; Simard et al. 2006; Hamilton 2013). To gain a better insight into the interactions of compound 5 with serum albumins molecular docking and dynamics analysis were carried out. Four different algorithms were used to find the binding site of compound 5 on serum albumins: AutoDock 4.2, AutoDock Vina, PatchDock/FireDock and SwissDock. These programs use different approaches to model the ligand protein interactions, such as, PatchDock uses shape complementarity whereas AutoDock 4.2 uses genetic algorithm. Molecular docking analysis by all these four programs shows that the interactions of the compound 5 with serum albumins were thermodynamically favorable (Table 4). The binding free energies computed by AutoDock Vina and SwissDock are very similar to that of the experimentally obtained values (Table 2). Molecular docking also provides the insight into the most favorable binding site for these compounds on the serum albumins (Fig. 4). The binding sites for the compound lie in the groove between domain I and domain III of BSA, whereas it is within the domain I in case of HSA (Fig. 4). Thermodynamics analysis from temperature dependent quenching studies also suggest differential nature of interaction with BSA and HSA. Thus, the docking studies which produced two different binding sites for HSA and BSA also support the experimentally obtained results (Table 2). Figure 4 also compares the best binding poses obtained by four different docking algorithms. PatchDock is a rigid docking algorithm and, therefore, AutoDock 4.2, Vina and SwissDock predicted ligand poses were used as input ligand structures for PatchDock. PatchDock results suggests that vina outputs were the best solution among AutoDock 4.2, Vina and SwissDock (Fig. 4). AutoDock Vina results, therefore, were used for further analysis and in molecular dynamics simulation.

**Table 4:** Theoretical binding free energies as obtained by molecular docking experiments using four different algorithms

Protein	AutoDock 4.2 (kJ mol^−1^)^a^	AutoDock Vina (kJ mol^−1^)	PatchDock/FireDock (kJ mol^−1^)	SwissDock (kJ mol^−1^)
BSA	−14.37 ± 0.36	−30.96	−47.85	−36.99
HSA	−17.34 ± 0.37	−33.47	−54.54	−33.22

^a^The data represent mean ± SEM

**Fig. 5:**
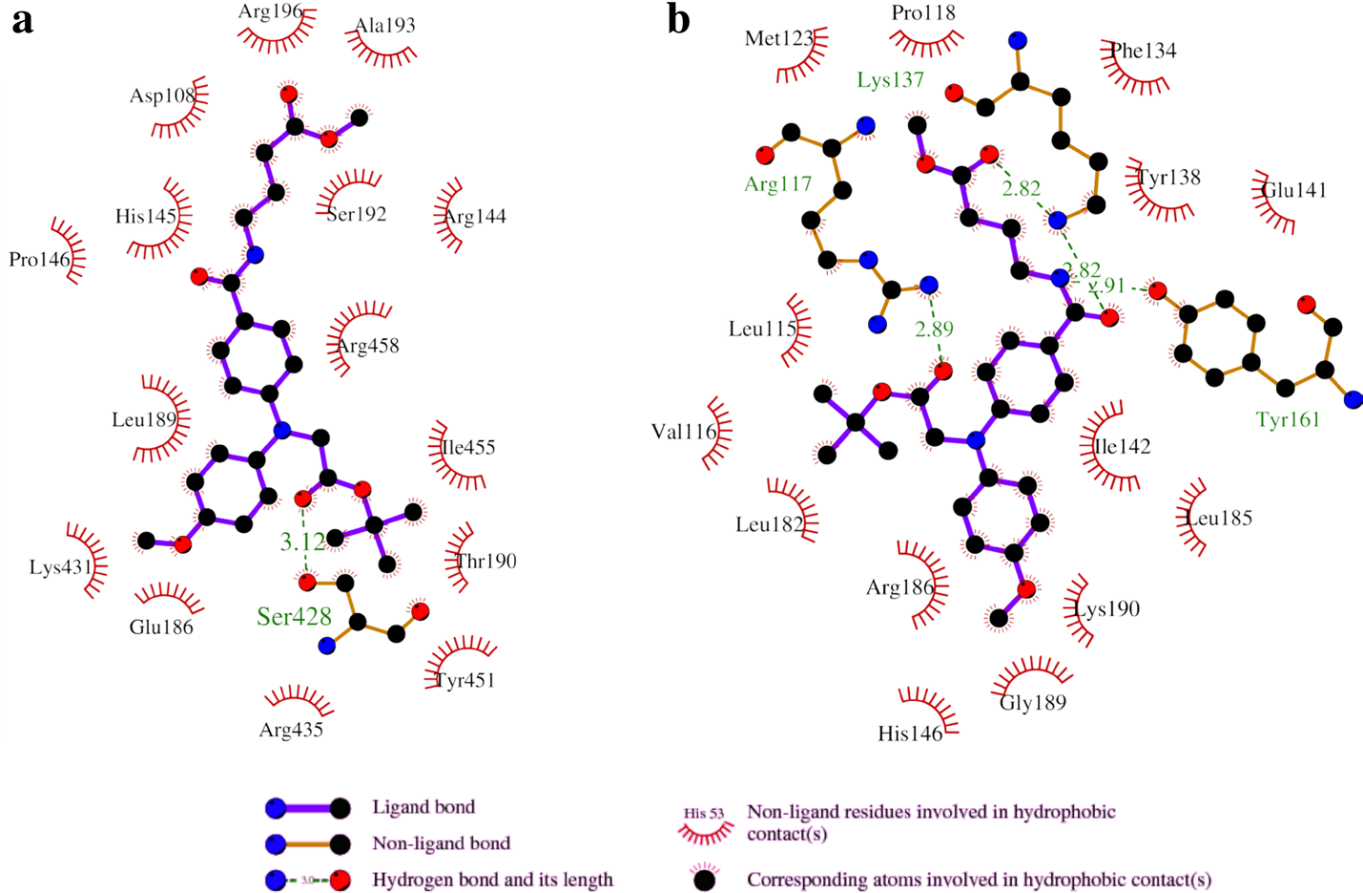
Detailed interaction of compound 5 with serum albumins. **a** 2D representation of the compound 5-BSA interaction diagram as obtained by LigPlot+. Hydrophobic and hydrogen bonding interactions and the interacting protein side chain residues are shown. **b** 2D representation of the compound 5-HSA interaction diagram as obtained by LigPlot+. Hydrophobic and hydrogen bonding interactions and the interacting protein side chain residues are shown

Figure 5 shows the detailed interaction diagram for the interaction of compound 5 with BSA and HSA. The figure shows that the interaction with BSA is mainly hydrophobic in nature, however, a hydrogen bond formation was observed with Ser428. Residues from domain I and domain III of BSA are involved in the interaction. On the other hand, interaction with HSA was mediated by four hydrogen bonds. The side chain NH group of Arg117 of HSA forms a hydrogen bond with the compound 5. Side chain NH of Lys137 was found to form two hydrogen bonds and the Tyr161 was also involved to form a hydrogen bond with the ligand through the phenolic OH group. Hydrophobic interactions also play a significant role in the interaction of compound 5 and HSA.

### Dynamics of compound 5 binding with serum albumins

**Fig. 6:**
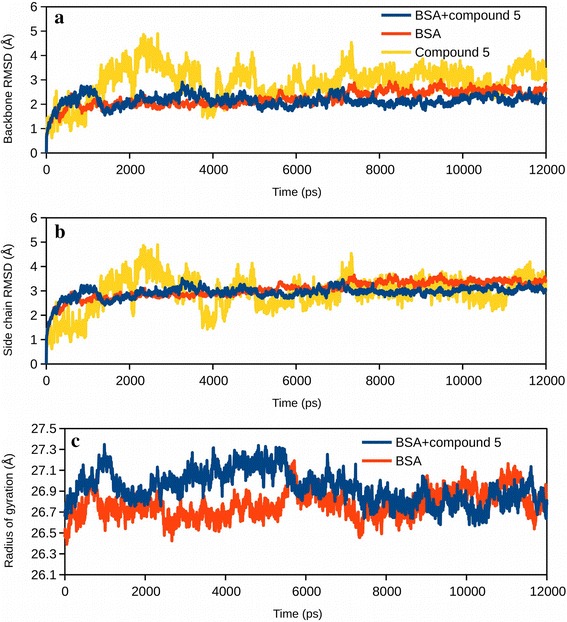
Molecular dynamics simulation of BSA-compound 5 complex. **a** Root mean square deviations (RMSD) of atomic positions of BSA backbone with respect to the initial structure, in presence (*blue*) and absence (*red*) of compound 5. Ligand RMSD with respect to BSA is also shown (*yellow*). **b** RMSD of atomic positions of BSA side chains with respect to the initial atom positions, in presence (*blue*) and absence (*red*) of compound 5. RMSD of compound 5 with respect to BSA is also shown. **c** Change in the radius of gyration of BSA with time in presence and absence of compound 5

**Fig. 7:**
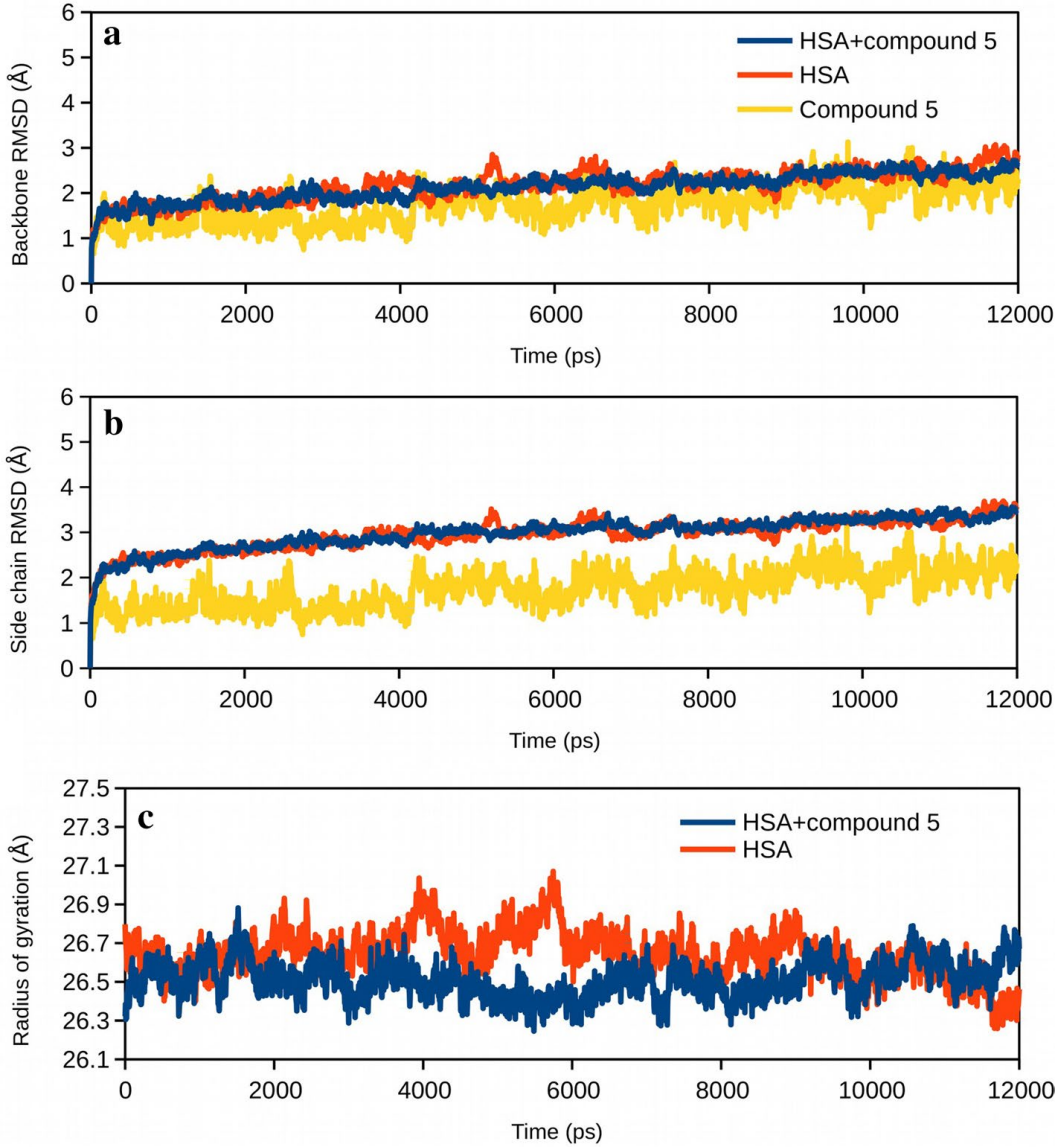
Molecular dynamics simulation of HSA-compound 5 complex. **a** RMSD of atomic positions of HSA backbone with respect to the initial structure, in presence (*blue*) and absence (*red*) of compound 5. RMSD of compound 5 with respect to HSA is also shown (*yellow*). **b** RMSD of atomic positions of HSA side chains with respect to the initial atom positions, in presence (*blue*) and absence (*red*) of compound 5. RMSD of compound 5 with respect to HSA is also shown. **c** Change in the radius of gyration of HSA with time in presence and absence of compound 5

**Fig. 8:**
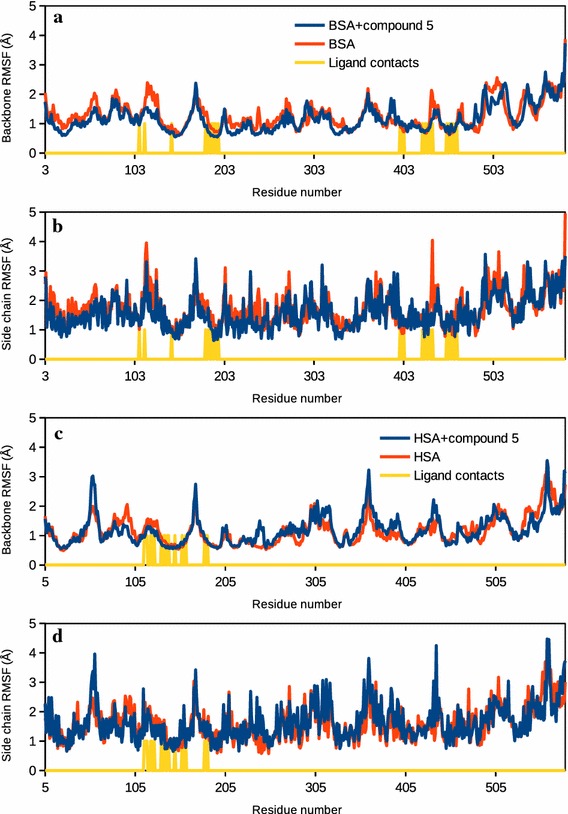
Fluctuations in the protein backbone and side chains when bound to compound 5. **a** Root mean square fluctuations (RMSF) in the backbone of BSA in presence (*blue*) and absence (*red*) of compound 5. Ligand contact sites are shown with *vertical yellow lines*. **b** RMSF in the side chains of BSA in presence and absence of compound 5. Ligand contact sites are shown. **c** RMSF in the backbone of HSA in presence and absence of compound 5. Ligand contact sites are shown. **b** RMSF in the side chains of HSA in presence and absence of compound 5. Ligand contact sites are shown

Molecular dynamics analysis was carried out to further investigate the stability of the complex formation. It also allowed us to observe the effect of protein side chain flexibility in the binding site as well as the effect of binding on the overall structure of the protein. Figure 6, 7, 8, Additional files 2 and 3 summarize the changes observed during the 12 ns time scale of molecular dynamics simulation. Figure 6a, b shows the root mean square deviation (RMSD) plots for the BSA and its complex with the ligand. Changes in the RMSD values indicated the protein is undergoing a conformational change. However, changes of the order of 1–3 Å are negligible for small, globular proteins. RMSD changes also suggests that the simulation has converged very rapidly and the protein/complex reached a stable conformation after around 1 ns. Figure 6c shows the changes in the radius of gyration of the protein in presence and in absence of the compound. The radius of gyration is an indicator of the compactness of the protein. Initially, in presence of the ligand BSA showed slightly relaxed conformation (Fig. 6c), however, the structure converged after about 5 ns and attained a more compact conformation. Figure 7 also shows the changes in RMSD and the radius of gyration of HSA in presence and in absence of the compound.

Figure 8, on the other hand, highlights the residue-wise fluctuations in the backbone and the side chains of BSA and HSA. The ligand contact sites are also highlighted. Figure 8 suggests that the backbone fluctuation slightly decreased near the ligand binding site, both, in BSA and in HSA. Decrease in the backbone fluctuations near the ligand indicated that the binding site attained a stable conformation. A video of the dynamics of BSA-compound 5 complex is shown in Additional file 2: Video S1. Additional file 3: Video S2 shows the dynamics of HSA-compound 5 in water.

**Fig. 9:**
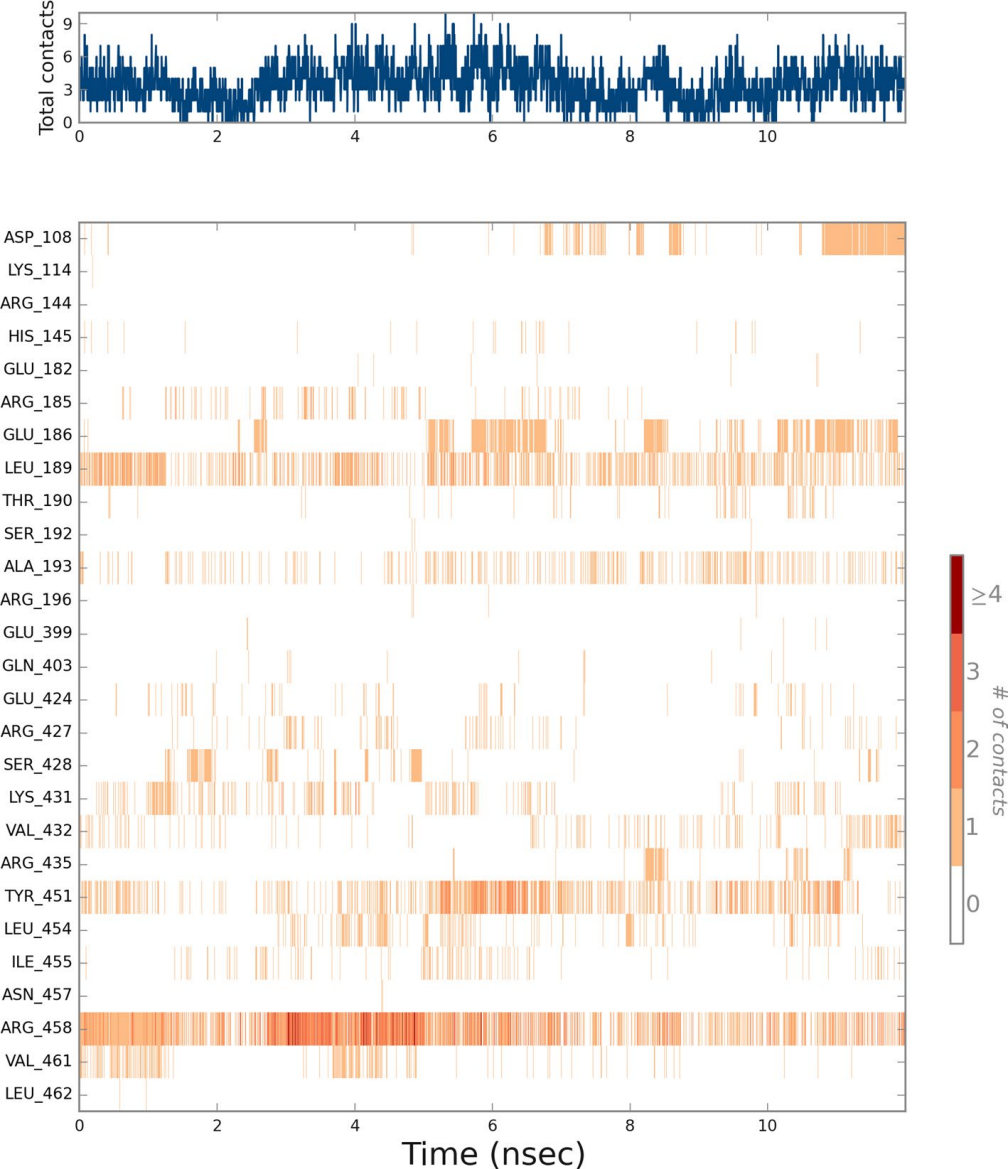
Ligand contacts with BSA over the time frame of molecular dynamics simulation. Number of hydrogen bonds, hydrophobic, ionic interactions and water bridges are measured. The *top panel* shows the total number of specific contacts BSA makes with compound 5 over the course of the trajectory. The *bottom panel* shows which residues of BSA interact with compound 5 in each trajectory frame. Some residues make more than one specific contact with the ligand, which is represented by a *darker shade of orange*, according to the scale to the *right* of the *plot*

**Fig. 10:**
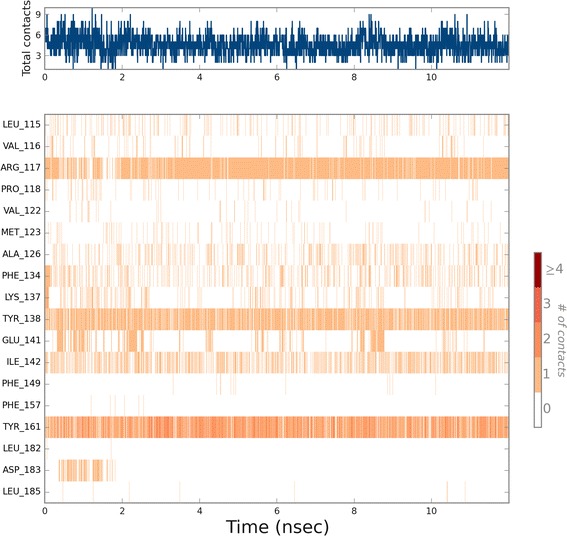
Ligand contacts with HSA over the time frame of molecular dynamics simulation. Number of hydrogen bonds, hydrophobic, ionic interactions and water bridges are measured. The *top panel* shows the total number of specific contacts HSA makes with compound 5 over the course of the trajectory. The *bottom panel* shows which residues of HSA interact with compound 5 in each trajectory frame. Some residues make more than one specific contact with the ligand, which is represented by a *darker shade of orange*, according to the scale to the *right* of the *plot*

Over the course of simulation, the ligand makes stable as well as transient contacts with the surrounding residues at the binding site. Such ligand contacts over the simulation time scale are depicted in Figs. 9 and 10. Figure 9 summarizes the contacts with BSA. From this figure it appears that Arg458 of BSA forms most contacts with the ligand. It forms hydrogen bonds, ionic interactions as well as water bridges with the compound 5. Other residues that were fund to form hydrogen bonds were Leu189 and Ser428. Tyr451 also maintained a consistent hydrophobic contact with the compound. Figure 10 summarizes the contacts with HSA. The residues that maintained consistent contacts over the simulation were Tyr161, Arg117 and Tyr138. The first two form predominantly hydrogens bonds whereas the Tyr138 provides hydrophobic environment.

## Conclusion

We have reported here, the synthesis and physicochemical properties of a new derivative of the naturally occurring inhibitory neurotransmitter GABA and its interactions with the drug carrier protein in blood, the serum albumin. Binding of this molecule with two orthologs of serum albumins, human and bovine, were compared. The compound shows drug like properties and binds to the human and bovine serum albumins with the binding constants in low micromolar concentration range. Thermodynamics analysis showed that the binding of compound 5 with HSA was enthalpy driven, whereas binding with BSA was driven by entropy. Molecular docking studies by various different algorithms further showed that the compound binds to the groove between domain I and domain III of BSA and within the domain I in case of HSA. Molecular dynamics analysis showed that the compound forms stable complexes with the serum albumins. Binding of the compound with BSA was stabilized mainly by hydrophobic and ionic interactions, whereas, interactions with HSA was maintained predominantly through hydrogen bonding.

## Additional files

10.1186/s40064-016-2752-x Detailed procedure of the synthesis of methyl 4-(4-((2-(*tert*-butoxy)-2-oxoethyl)(4-methoxyphenyl)amino)benzamido)butanoate and the characteristic data. ^1^H and ^13^C NMR spectra of the compound are also given in the Additional file 1. It also contains Figure S1 and Table S1.
10.1186/s40064-016-2752-x The dynamics of BSA-compound 5 in water over the course of 12 ns time is shown in Video S1.
10.1186/s40064-016-2752-x The dynamics of HSA-compound 5 in water over the course of 12 ns time is shown in Video S2.
