# Supplementary material for: Binding interaction of a gamma-aminobutyric acid derivative with serum albumin: an insight by fluorescence and molecular modeling analysis
Source: Springerplus. 2016 Jul 19;5(1):1121. doi: 10.1186/s40064-016-2752-x (PMC4949196; doi:10.1186/s40064-016-2752-x)
Supplement: Supplementary file 1 — 10.1186/s40064-016-2752-x Detailed procedure of the synthesis of methyl 4-(4-((2-(tert-butoxy)-2-oxoethyl)(4-methoxyphenyl)amino)benzamido)butanoate and the characteristic data. 1H and 13C NMR spectra of the compound are also given in the Additional file 1. It also contains Figure S1 and Table S1. [file 40064_2016_2752_MOESM1_ESM.pdf]

Additional file 1:

Binding interaction of a gamma-aminobutyric acid derivative with serum albumin: An insight by fluorescence and molecular modeling analysis

Uttam Pal[a, 1], Sumit Kumar Pramanik[b, 1], Baisali Bhattacharya[a], Biswadip Banerji[b], Nakul Chandra Maiti[a, *]

[a] *Structural Biology & Bioinformatics Division, Council of Scientific & Industrial Research (CSIR)-Indian Institute of Chemical Biology (IICB), Kolkata, West Bengal, India*

[b] *Chemistry Division, Council of Scientific & Industrial Research (CSIR)-Indian Institute of Chemical Biology (IICB), Kolkata, West Bengal, India*

* Corresponding author. Tel.: +91 33 2499 5940; fax: +91 33 2473 5197.

E-mail address: ncmaiti@iicb.res.in (N.C. Maiti).

[1] These authors contributed equally to this work.

Detailed procedure of synthesis of the GABA derivative:

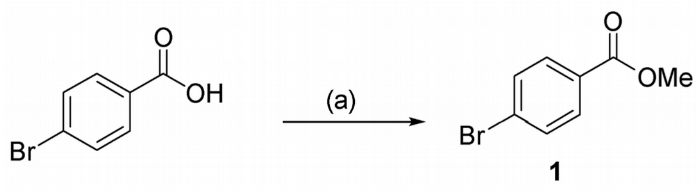

Scheme 1 Reagent and conditions: (a) Conc. H_2SO_4, 0 °C to rt, 6 hrs

To a stirred solution of 4-bromobenzoic acid (1 equiv.) in methanol was added H_2SO_4 (10 % by mass) at 0 °C. The reaction was then refluxed for 6 hours. The reaction mixture was then neutralized by 1 M NaOH solution and extracted with ethyl acetate. The ethyl acetate was then concentrated to dryness, and the residue was purified by column chromatography (hexane/ ethyl acetate) to afford **1**.

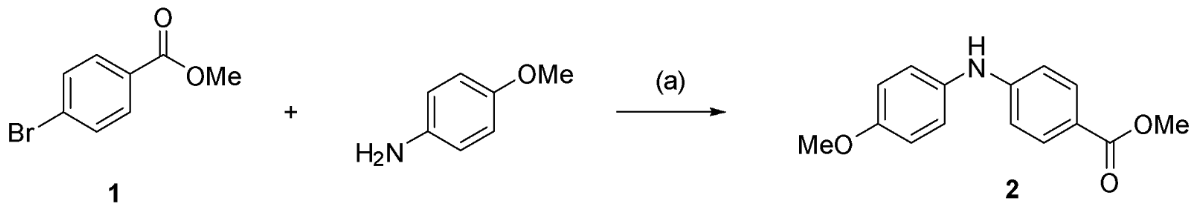

Scheme 2 Reagent and conditions: (a) palladium (II) acetate (0.05 equiv.), xantphos (0.1 equiv.) and cesium carbonate (3 equiv.), 80 °C, 4 hrs.

To a stirred solution of compound **1** (1 equiv.) and 4-methoxyaniline (1.2 equiv.) in 1,4-dioxane was added palladium(II) acetate (0.05 equiv.), xantphos (0.1 equiv.) and cesium carbonate (3 equiv.). The reaction was then continued at 80 °C for 4 hours. The reaction mixture was filtered, concentrated to dryness, and the residue was purified by column chromatography (hexane/ ethyl acetate) to afford **2**.

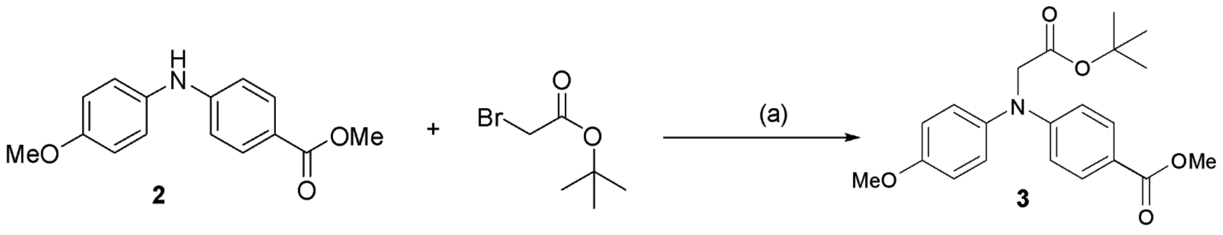

Scheme 3 Reagent and conditions: (a) potassium tert-butoxide (1.2 equiv.), DMF, 0 °C to rt, 12 hrs.

To a stirred solution of compound **2** (1 equiv.) in dimethyl formamide was added tert-butyl bromo acetate (1.2 equiv.) and potassium tert-butoxide (1.2 equiv.) at 0 °C. The reaction was then continued at room temperature for 12 hours. The reaction mixture was worked up with ethyl acetate and water. The ethyl acetate was concentrated to dryness, and the residue was purified by column chromatography (hexane/ ethyl acetate) to afford **3**.

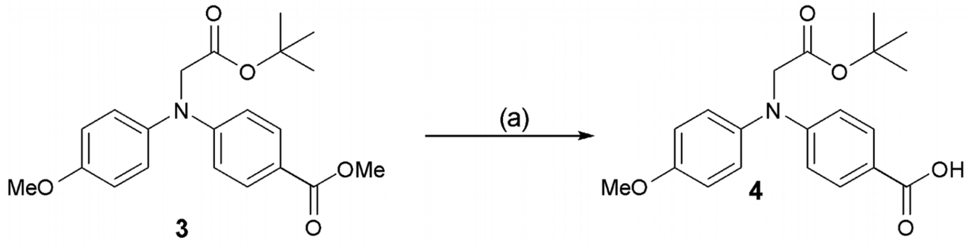

Scheme 5 Reagent and conditions: (a) Lithium hydroxide (3 equiv.), MeOH-water (5:1), r.t., 2 hrs..

To a stirred solution of compound **3** (1 equiv.) in MeOH-water (5:1) was added lithium hydroxide (3.0 equiv.) at 0 °C. The reaction was then continued at room temperature for 1.5 hours. MeOH was then evaporated and the solution was then neutralized by using 1 M HCl solution and the compound was then extracted with ethyl acetate. The ethyl acetate was then concentrated to dryness to afford **4**, which was carried out to the next step without further purification.

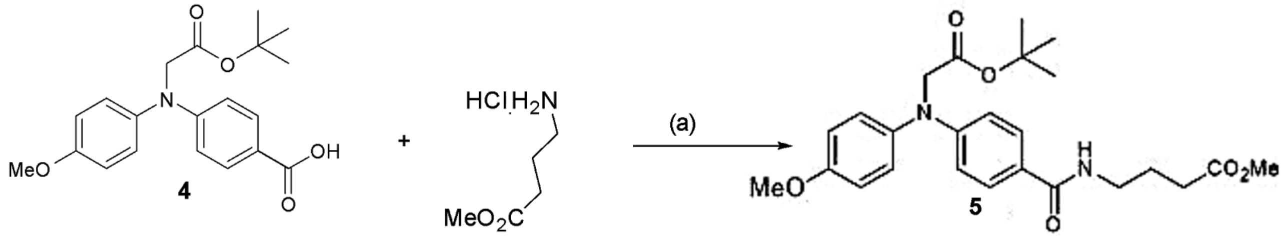

Scheme 6 Reagent and conditions: (a) EDC.HCl (1.5 equiv.), HOBT (1.2 equiv.), TEA (3 equiv.), 0 °C to rt, 1.5 hrs.

To a stirred solution of compound **4** (1 equiv.) and methyl 3-aminopropanoate hydrochloride (1.2 equiv.) in dry THF was added EDC.HCl (1.5 equiv.), HOBT (1.2 equiv.) and triethyl amine (3 equiv.) at 0 ºC. The reaction was then continued at room temperature for 7 hours. The reaction mixture was concentrated to dryness, and the residue was purified by column chromatography (hexane/ ethyl acetate) to afford **5**.

Characteristic data

Methyl 4-(4-((2-(tert-butoxy)-2-oxoethyl)(4-methoxyphenyl)amino)benzamido)butanoate:

Light yellow solid, m.p.= 199-200°C, 1H NMR (600 MHz, CDCl3): δ (in ppm) 1.44 (9 H, s), 1.95-1.91 (2 H, m), 2.42 (2 H, t, J = 7.2), 3.48-3.45 (2 H, m), 3.66 (3 H, s), 3.82 (3 H, s), 4.20 (2 H, s), 6.33 (1 H, t, J = 4.8), 6.55 (2 H, d, J = 9.0), 6.95-6.92 (2 H, m), 7.26-7.23 (2 H, m), 7.69 (2 H, d, J = 9.0); 13C NMR (150 MHz, CDCl3): δ (in ppm) 24.58, 28.02, 31.71, 39.42, 51.74, 55.13, 55.46, 82.02, 112.69, 115.04, 123.02, 128.11, 128.78, 139.18, 150.88, 157.84, 167.27, 169.48, 174.29; HRMS (FAB+): (m+H) / z Calcd for C25H33N2O6 (M+H)+ 457.2339, Found: (m+H) / z 457.2339

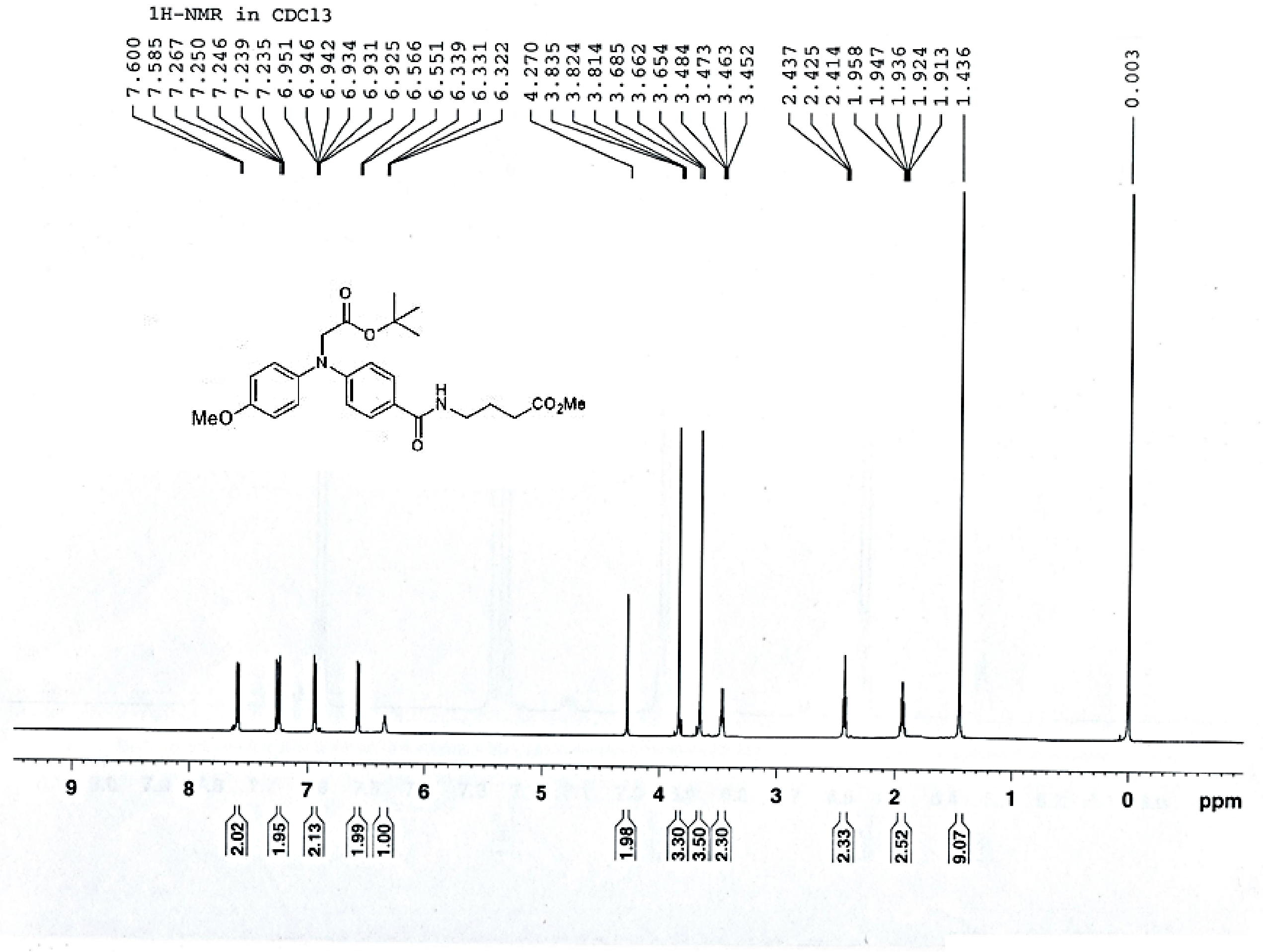

^{1}H NMR in $CDCl_3$

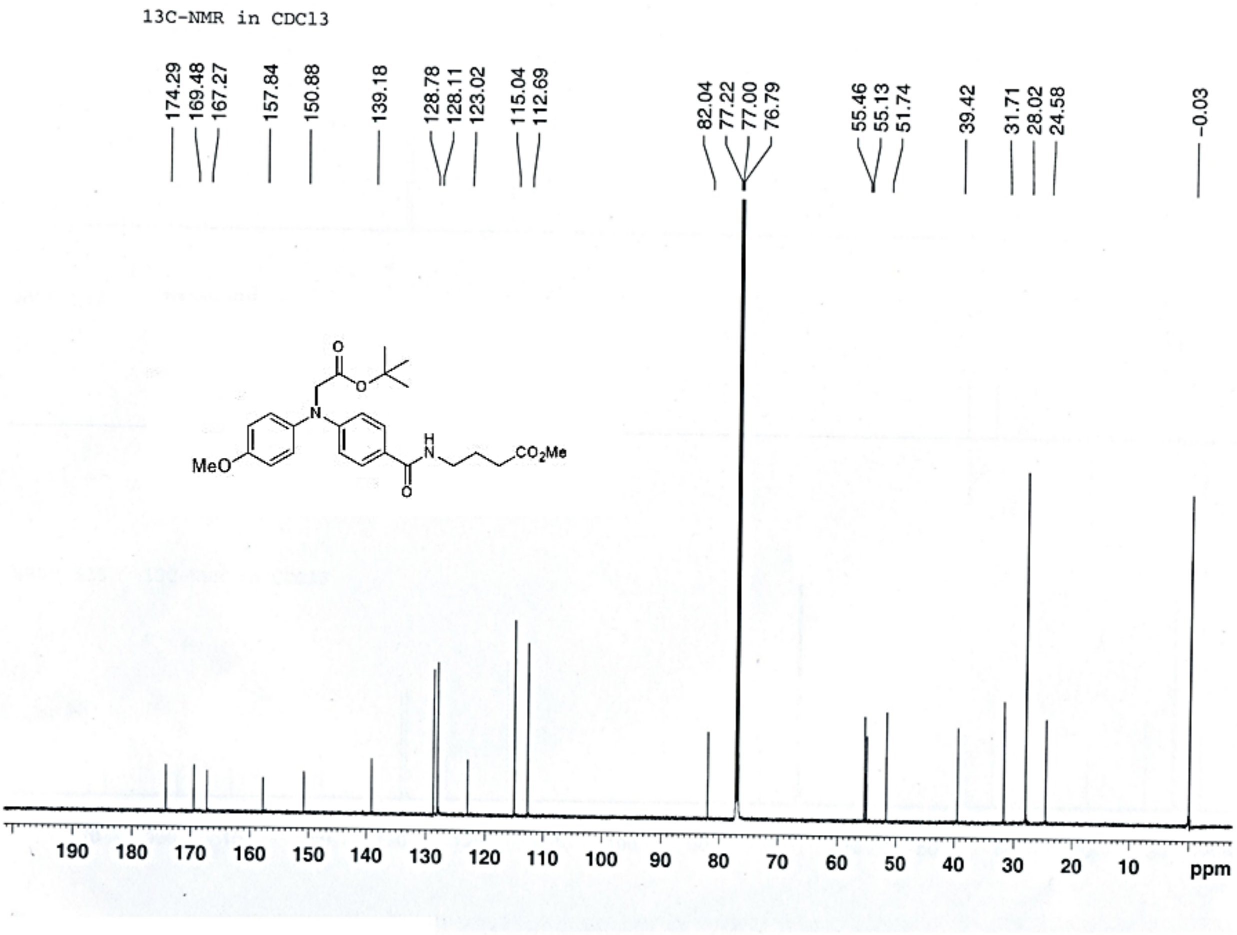

^{13}C NMR in $CDCl_3$

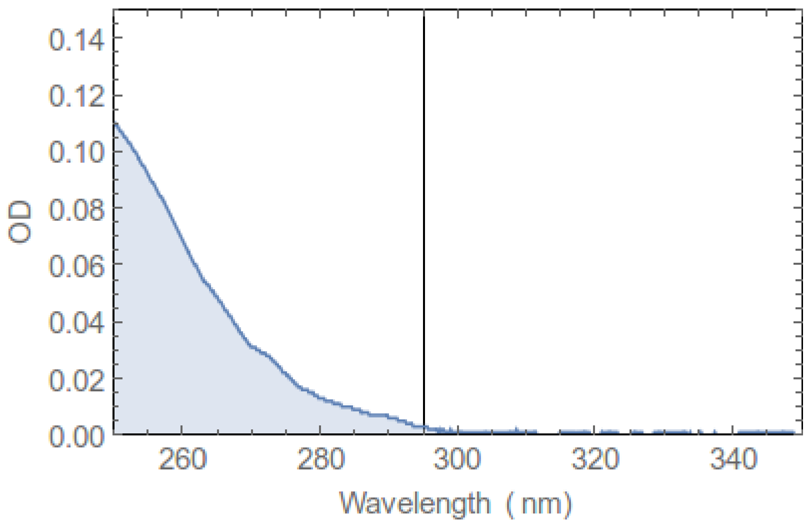

Figure S1: Absorption behavior of compound 5 in the UV-Visible range. Absorption spectrum of 5 μM of compound in 20 mM Tris-HCl buffer of pH 7.0.

Table S1 Global cell parameters for molecular dynamics in Desmond. x, y and z components of the a, b anc c vectors of the triclinic simulation box is given.[#]

Components	BSA complex	BSA	HSA complex	HSA
ax	91.29	94.37	94.81	94.81
ay	0	0	0	0
az	0	0	0	0
bx	38.29	49.16	39.27	39.27
by	66.31	85.14	68.02	68.02
bz	0	0	0	0
cx	52.1	39.42	47.76	47.76
cy	30.08	22.76	27.57	27.57
cz	85.07	64.37	77.99	77.99

[#]Values are in agnstroms.

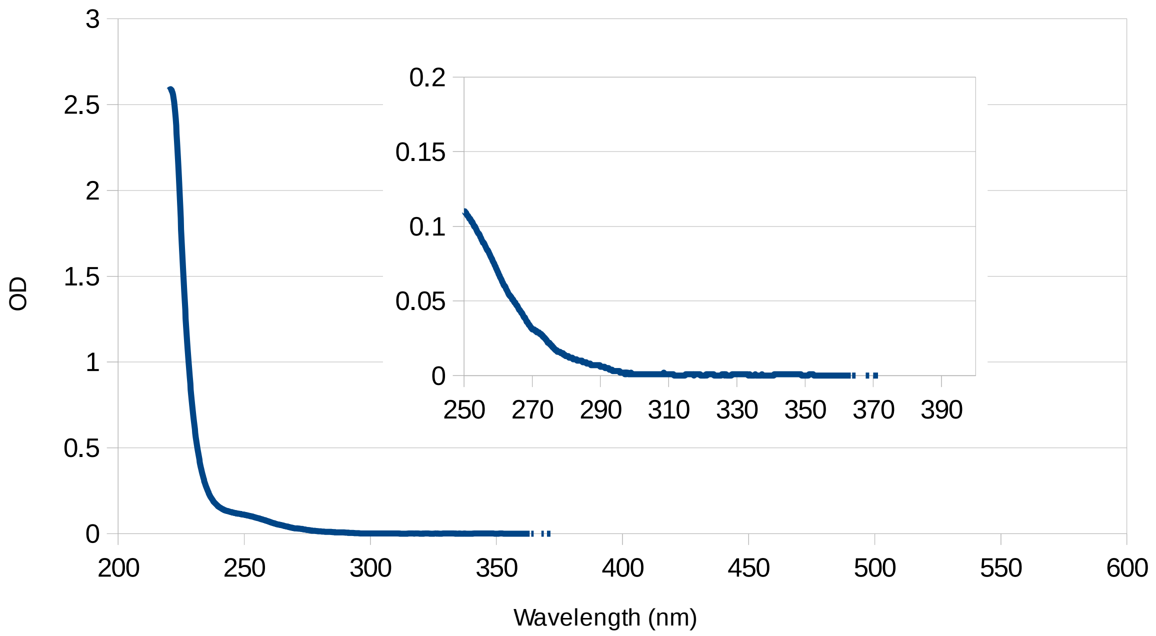

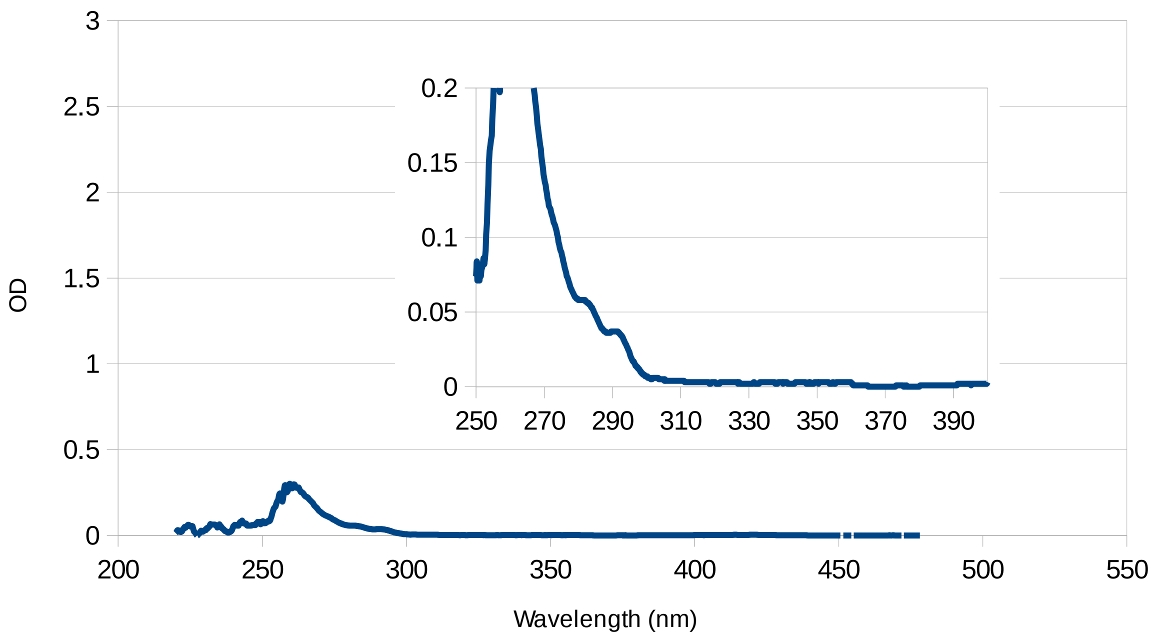

Figure S2: Full length absorption spectra of compound 5 in 20 mM TrisHCl buffer, pH 7.0 (top) and in DMSO (bottom). Compound 5 concentration was ~5 μM.

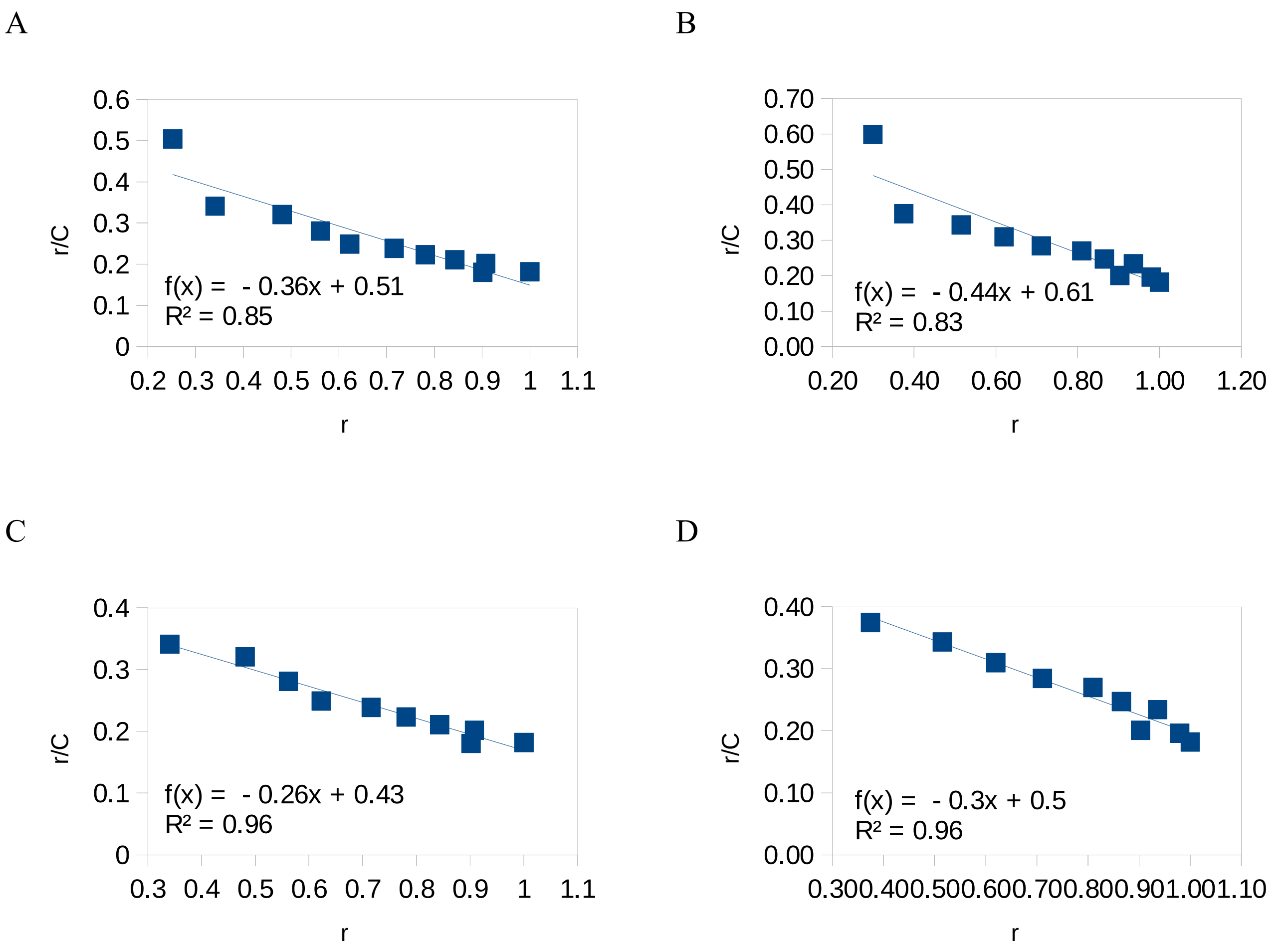

Figure S3: Scatchard plot. (A) Binding stoichiometry for BSA was found to be 1.46±0.06. (B) Binding stoichiometry for HSA was found to be 1.46±0.03. (C) Considering the first point in plot A as outlier, binding stoichiometry for BSA was found to be 1.65. (D) Considering the first point in plot A as outlier, binding stoichiometry for HSA was found to be 1.63. The data for this plot was derived from the spectra shown in Figure 1. r= $\Delta F/\Delta F_{max}$, C is the concentration of ligand.

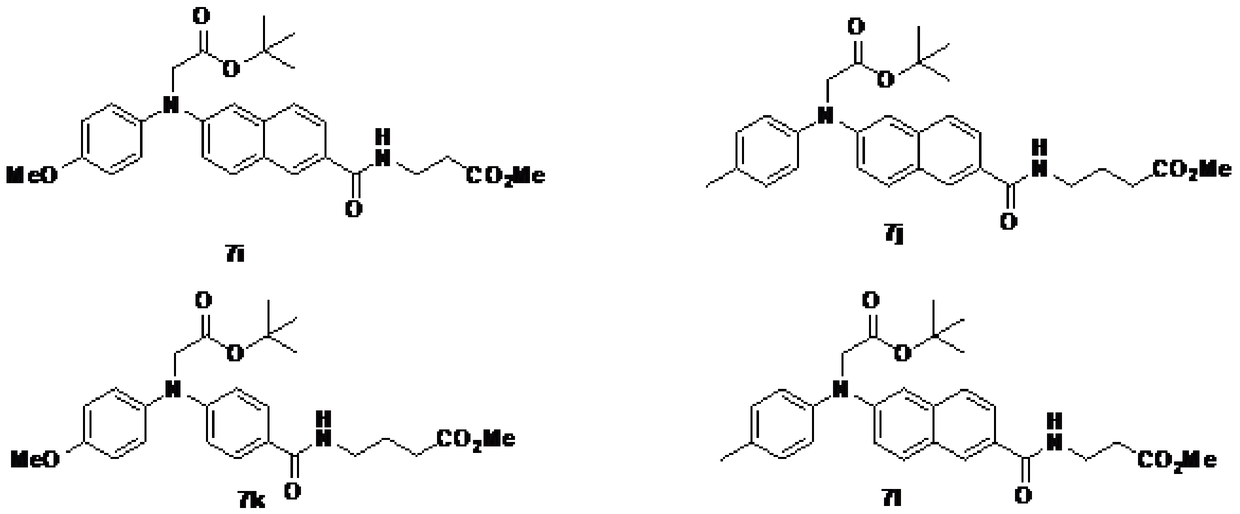

Figures S4: Some of the compounds in the series developed to test their anti-Alzheimer's properties (Sanphui et al. 2013). The photophysical properties of compound 7i and its binding with serum albumins were reported earlier (Pal et al. 2015). Here, in this article we reported the serum albumin binding of 7k (compound 5). 7l does not show good binding with serum albumin as shown in the following figure.

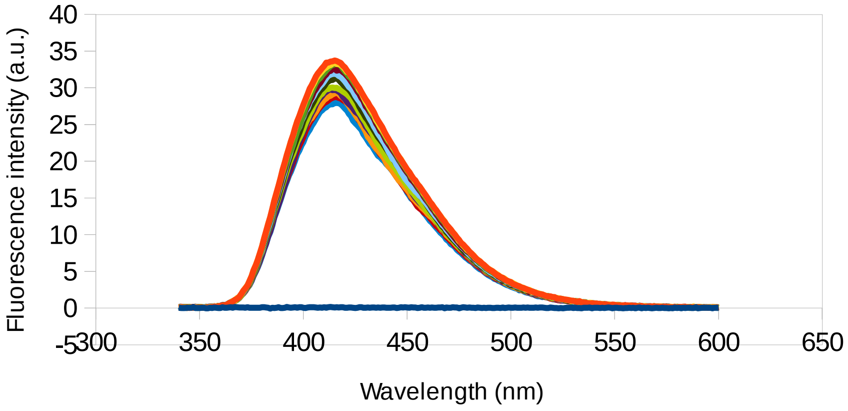

Figure S5: Unlike 7i (Pal et al. 2015), 7l shows fluorescence quenching with increasing concentrations of BSA. However, the quenching efficiency is very low.
